# Efficacy of Oral Etoposide in Pretreated Metastatic Breast Cancer: A Multicenter Phase 2 Study

**DOI:** 10.1097/MD.0000000000000774

**Published:** 2015-05-01

**Authors:** Peng Yuan, Lijun Di, Xiaohui Zhang, Min Yan, Donggui Wan, Li Li, Yongqiang Zhang, Jufen Cai, Hong Dai, Qi Zhu, Ruoxi Hong, Binghe Xu

**Affiliations:** From the Department of Medical Oncology (PY, RH, BX), Cancer Institute and Hospital, Chinese Academy of Medical Sciences and Peking Union Medical College; Peking University Cancer Hospital (LD); Peking Union Medical College Hospital (XZ), Beijing; Henan Cancer Hospital (MY), Zhengzhou; China-Japan Friendship Hospital (DW), Beijing; First Affiliated Hospital of Dalian Medical University (LL), Dalian; Beijing Hospital of the Ministry of Health (YZ); Zhejiang Cancer Hospital (JC); Beijing ChaoYang Hospital (HD), Beijing; and Shanghai Putuo District People's Hospital (QZ), Shanghai, China.

## Abstract

No standard chemotherapy has been defined for metastatic breast cancer (MBC) patients pretreated with anthracyclines and taxanes. A multicenter phase 2 study was conducted to evaluate the safety and efficacy of oral etoposide in patients with MBC.

Eligible patients were treated with repeated cycles of oral etoposide (60 mg/m^2^/d on days 1–10, followed by 11 days of rest). The primary endpoint was progression-free survival (PFS). The secondary endpoints were objective response rate, clinical benefit rate (CBR), and toxicity profiles.

Seventy-five women with MBC were enrolled at 10 centers in China. Seven (9.3%) patients achieved partial response (PR) and 29 (38.7%) had stable disease (SD). Nine patients (12%) had SD for >24 weeks and the CBR was 21.3% (16/75). The median PFS was 4.5 (range, 1.3–7.7) months. Of the 38 patients who received ≥3 regimens prior to this study, 2 (5.3%) had PR and 3 (7.9%) had SD for >24 weeks, with a CBR of 13.2%. The reported grade 3/4 adverse events included leukopenia (13.3%, n = 10), neutropenia (17.9%, n = 14), anemia (2.7%, n = 2), vomiting (2.6%, n = 2), and alopecia (1.3%, n = 1).

Oral etoposide was effective and well tolerated in Chinese women with pretreated MBC.

## INTRODUCTION

Significant and worldwide progress in the early detection and comprehensive treatment of breast cancer (BC) has been made in recent years. However, it has been estimated that 30% of the patients initially diagnosed with early-stage BC will eventually develop metastatic breast cancer (MBC). In most cases, MBC remains an incurable disease. Therefore, the systemic treatment of MBC prolongs survival and enhances quality of life, but is not curative.

Anthracyclines and taxanes are the preferred cytotoxic drugs for the treatment of MBC. Other agents, including capecitabine, gemcitabine, vinorelbine, and others, are also available as treatment options and can be used in cases that were pretreated with at least an anthracycline and a taxane.^1–5^ For patients who have failed ≥3 chemotherapy regimens, there are no standard therapeutic schedules. Most guidelines suggest best supportive therapy or participation in clinical trials.

Etoposide is a semisynthetic derivative of podophyllotoxin that can induce cell cycle arrest at the late S phase and early G2 phase.^6^ Although it has been administered intravenously to MBC patients with disappointing results,^7^ a number of preclinical and clinical studies have demonstrated the schedule dependency of etoposide efficacy.^8–10^ In a phase 1 clinical trial, a variety of solid tumors showed responses to prolonged etoposide exposure.^11^ Hainsworth et al^11^ proposed a schedule of chronic oral etoposide of 50 mg/m^2^/d for 21 consecutive days every 4 weeks. Additionally, sensitivity to oral etoposide has been demonstrated in small cell lung cancer, nonsmall-cell lung cancer, non-Hodgkin lymphoma, germ cell tumors, and BC.^12,13^ Furthermore, the prolonged schedule of administration of single-agent oral etoposide has been tested in recurrent or MBC in five phase 2 clinical trials.^14–18^ The numbers of patients enrolled and the response rates in these studies are listed in Table 1. It is important to note that all of these studies included small sample sizes and were conducted before granulocyte colony-stimulating factor was used in clinical practice. Additionally, there is a lack of data pertaining to the efficacy and safety of oral etoposide in Chinese patients with MBC. In 2012, we reported our single-center study of oral etoposide in a group of Chinese patients with heavily treated MBC, the results of which suggested that oral etoposide could be an option for this patient population.^19^ To further elucidate the efficacy of oral etoposide, we conducted a multicenter phase 2 study in Chinese patients with MBC who were pretreated with anthracyclines and taxanes.

**TABLE 1:**
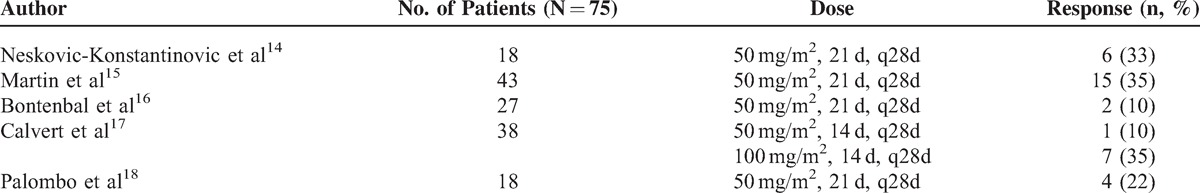
Phase 2 Clinical Trials of Oral Etoposide in Metastatic Breast Cancer

## METHODS

### Patients

Patients were eligible to participate in the study if they had histologically or cytologically confirmed BC with clinical evidence of metastatic disease. Patients were allowed to have undergone no >3 prior chemotherapy regimens for metastatic disease. Prior therapy must have included an anthracycline and a taxane in either the adjuvant or metastatic settings.

Additionally, patients must have had a life expectancy of at least 3 months, an Eastern Cooperative Oncology Group performance status of ≤ 2, and at least 1 measurable lesion. Other eligibility criteria included being between 18 and 80 years of age, and adequate bone marrow, liver, and renal function.

The trial was conducted in accordance with the principles of Good Clinical Practice and the Declaration of Helsinki. The study protocol was approved by the local ethics committee, and all patients provided written informed consent before any study-related procedure (ClinicalTrials.gov identifier: NCT01492556).

### Study Design

This was a single-arm, phase 2, multicenter study of single-agent oral etoposide as salvage treatment in pretreated patients with MBC. The primary objective was to evaluate progression-free survival (PFS), defined as the time from etoposide treatment initiation to progressive disease (PD) or death from any cause. Secondary objectives included assessing the objective response rate (ORR), clinical benefit rate (CBR), safety, and toxicity associated with etoposide treatment. ORR was defined as complete response (CR) and partial response (PR). Objective responses, stable disease (SD), and PD were evaluated according to the Response Evaluation Criteria in Solid Tumors (RECIST) criteria. CBR was defined as CR + PR + SD ≥24 weeks.

### Treatment Plan

Etoposide was administrated orally at a single dose of 60 mg/m^2^/d for 10 consecutive days every 3 weeks until confirmed PD or intolerable toxicity. If grade 3 hematologic toxicity and/or grade 2 nonhematological toxicity occurred during etoposide administration, the dose would be reduced to 75% in the following courses of therapy; after the second episode of grade 3 hematological toxicity and/or grade 2 nonhematological toxicity, the dose of oral etoposide would be reduced to 50%; upon the third episode of grade 3 hematologic toxicity and/or grade 2 nonhematological toxicity, or a second episode of grade 4 toxicity, the drug would be discontinued.

### Treatment Response and Toxicity Assessment

A baseline assessment, including medical history, physical examination, chest x-ray or computed tomography (CT), complete blood count, and full clinical chemistry tests was conducted in the patients prior to enrolment. In each cycle, whole blood cell counts and full clinical chemistry tests were conducted. Additionally, CT scanning and/or magnetic resonance imaging was applied after every 2 treatment cycles.

Tumor evaluation was performed after every 2 treatment cycles according to the RECIST criteria (version 1.1). Safety was assessed on the basis of reported adverse events and laboratory abnormalities. All toxicities were graded according to the National Cancer Institute Common Toxicity Criteria (version 4.0).

### Statistics

Intention-to-treat (ITT) analyses were performed for both efficacy and safety measures. Descriptive statistics were used to summarize the safety and laboratory observations. The median PFS and its accompanying 95% confidence interval were calculated using the Kaplan–Meier method and compared by log-rank tests.

## RESULTS

### Patient Population

In this study, 75 women with MBC were enrolled at 10 centers in China from September 2011 to April 2014. All these enrolled patients received at least 1 dose of etoposide treatment. After the treatment, the patients were included in the efficacy and safety analyses (ITT population). Two patients were withdrawn from the study because of the decline of further treatment after cycle 1. In fact, both showed SD after the first cycle of study treatment, as assessed by CT.

Baseline characteristics of the 75 eligible patients are presented in Table 2. The median age of the patients was 55 (range, 33–68) years with the median follow-up of 22.0 (range, 3.0–58.0) months. The majority of patients (69.0%) had visceral disease involving the lung and/or liver, and the cancer had spread to at least 2 distant sites in 78.0%. Half of the patients had received at least 2 chemotherapeutic regimens for MBC. More than 90% of the patients received anthracycline and/or taxane-containing regimens as adjuvant or metastatic therapy. Approximately 64% of the patients had received capecitabine and/or gemcitabine, 33.3% received a venorelbine-containing regimen, and 30.7% received platinums as metastatic therapy.

**TABLE 2:**
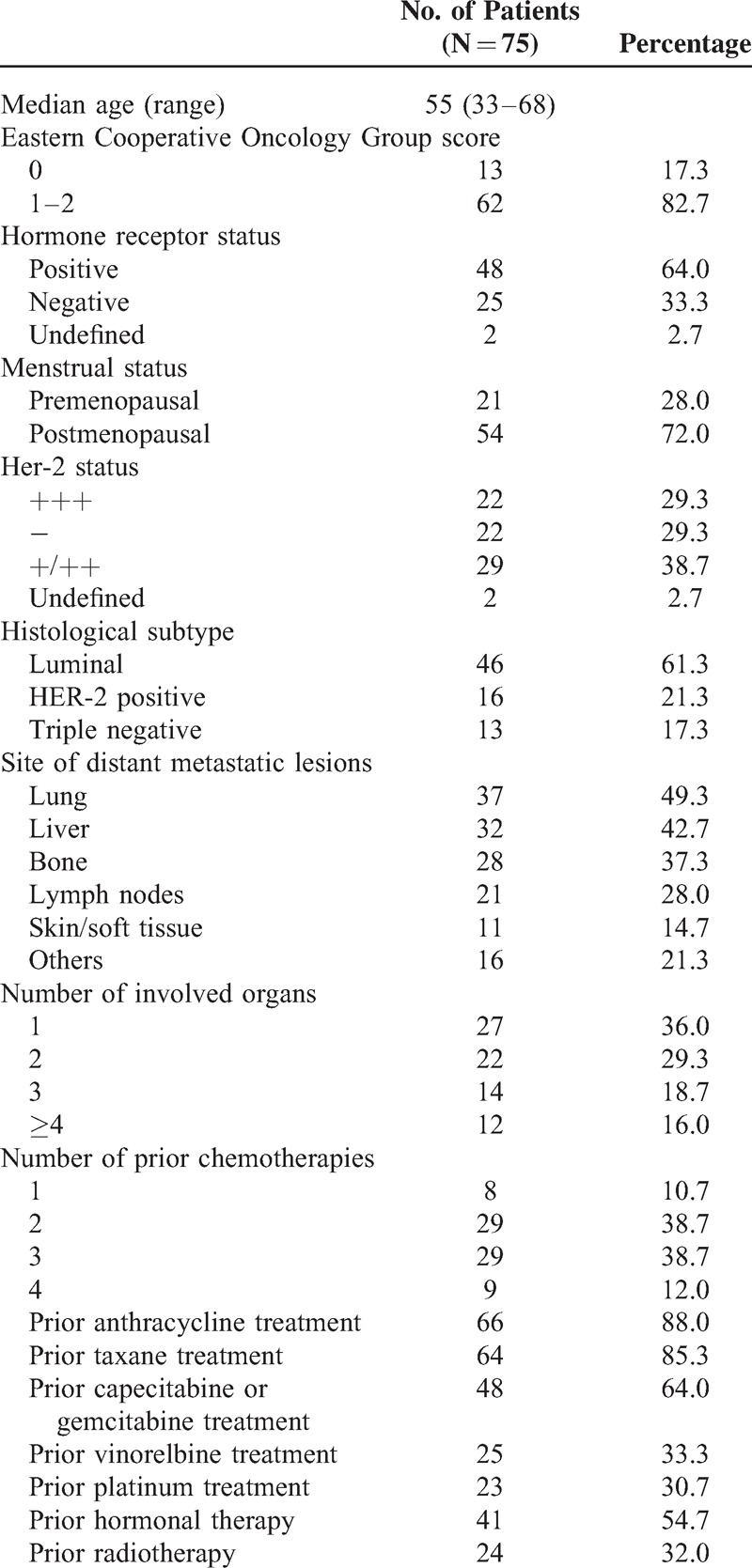
Patient Characteristics

### Treatment Exposure

A total of 417 treatment cycles were given, with a median of 4 (range, 1–44) cycles. Chemotherapy was delayed in 9 patients (12%) as a result of grade 3/4 neutropenia (n = 7) or liver dysfunction (n = 2). The chemotherapy dose was reduced in 6 patients (8%) because of febrile neutropenia (n = 2), grade 3 anemia (n = 2), or grade 3/4 vomiting (n = 2). A full dose intensity was achieved in 92% of patients (n = 69).

### Response and Survival

**FIGURE 1:**
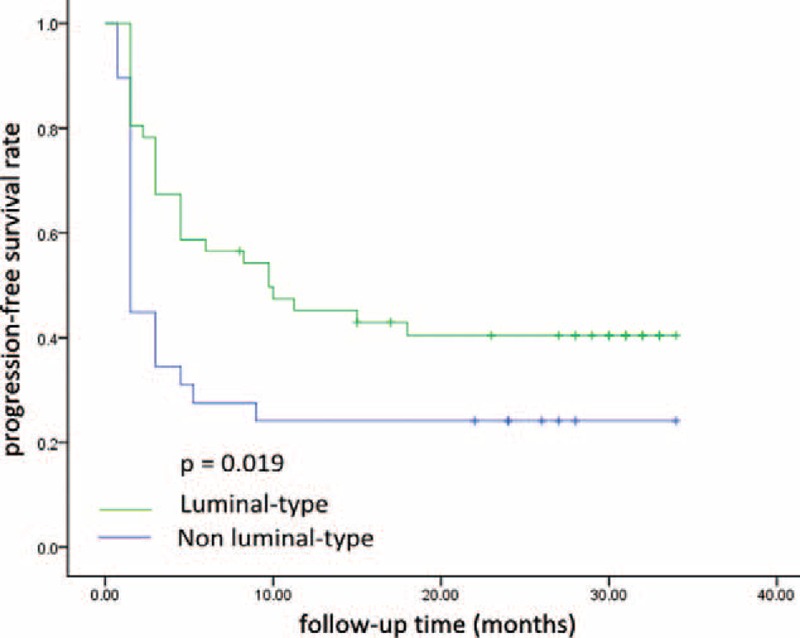
Progression-free survival (PFS) for patients with luminal-type breast cancer was significantly longer than those with nonluminal-type breast cancer (median PFS 9.7 vs 1.5 months, *P* = 0.019).

**FIGURE 2:**
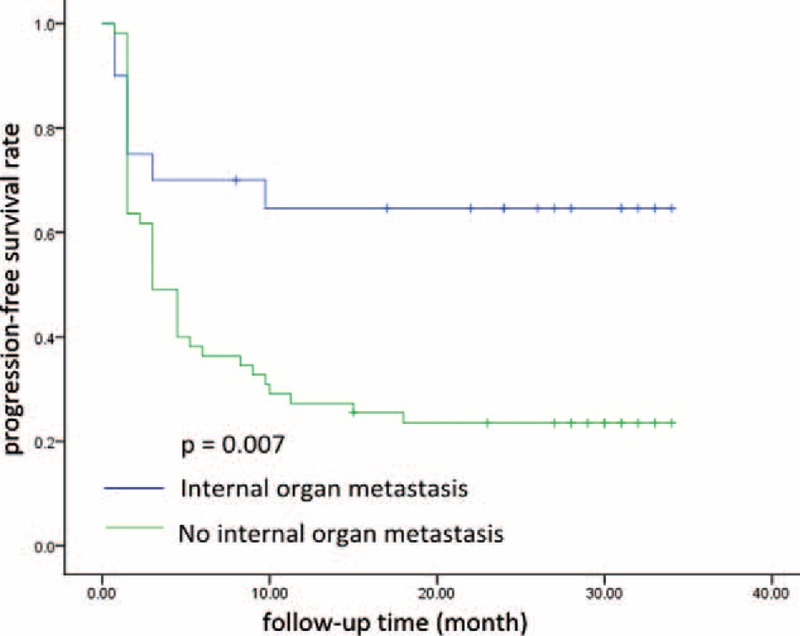
Patients who had internal organ metastases had significantly worse prognoses than those who did not (6.5 vs 3.0 months, respectively, *P* = 0.007).

No CR was observed. Seven of the 75 patients (9.3%) achieved PR, 29 (38.7%) achieved SD, 9 (12%) had SD for >24 weeks (long SD), and 39 (52.0%) experienced PD during treatment. The CBR was 21.3% (16/75). The median PFS was 4.5 (range, 1.3–7.7) months. Of the 38 patients who received ≥3 regimens prior to the current study, 2 (5.3%) had PR, 14 (36.8%) had SD, and 3 (7.9%) had long SD, with a CBR of 13.2%. The clinical responses for the overall patient population and for the subset of patients who had received ≥3 prior treatment regimens are shown in Table 3. Patients with luminal-type BC had higher CBR (25.5%) than those with HER-2 positive type and triple-negative BC (8.3% and 0.0%, respectively). PFS was also significantly longer for BC patients with luminal-type than those with nonluminal types (9.7 vs 1.5 months, *P* = 0.019, Figure 1). We also found that patients who had internal organ metastases had significantly worse prognoses than those who did not (6.5 vs 3.0 months, respectively, *P* = 0.007, Figure 2).

**TABLE 3:**
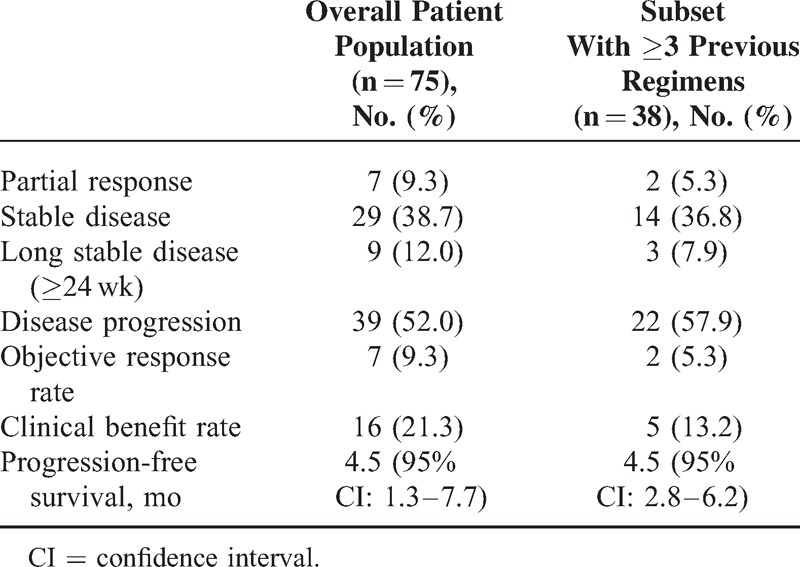
Clinical Responses in the Overall Patient Population (n = 75) and in the Subset of Patients Who Had Received ≥3 Prior Treatment Regimens

### Safety

Table 4 shows summarized hematological and nonhematological toxicities. No treatment-related deaths occurred. The most common hematologic adverse events were leukopenia (66.6%), neutropenia (60.6%), and anemia (24.1%). Nausea/vomiting were the most frequent nonhematological adverse events, with an incidence of 59.9%. Mucositis was observed in 12% (9/75) of the patients (grade 1, 6 patients; grade 2, 3 patients). The reported grade 3/4 adverse events included leukopenia (13.3%, n = 10), neutropenia (17.9%, n = 14), anemia (2.7%, n = 2), vomiting (2.6%, n = 2), and alopecia (1.3%, n = 1).

**TABLE 4:**
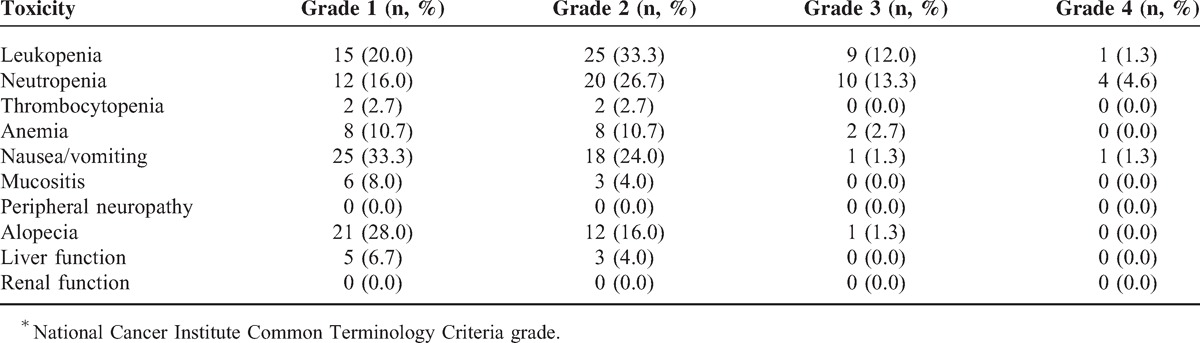
Hematological and Nonhematological Toxicities^∗^

## DISCUSSION

By forming a ternary complex with DNA and the topoisomerase II enzyme, etoposide catalyzes DNA topoform interconversions and introduces a DNA strand break.^20^ Furthermore, etoposide can induce single and double-strand DNA breaks even at low concentrations. However, this interaction is reversible after etoposide withdrawal. Therefore, prolonged cancer cell exposure to etoposide can achieve better cytotoxic activity. Consistent with these preclinical observations, the daily administration of oral etoposide has yielded optimal response rates in some tumors.

In the first reported phase 1 clinical trial of oral etoposide, Hainsworth et al^11^ demonstrated that etoposide could be tolerated up to 50 mg/m^2^/d, for 21 consecutive days of treatment followed by a 7-day break. This regimen has since been adopted by several investigators in treating MBC patients with a response rate between 10% and 33% depending on the number of prior treatment regimens. Neskovic-Konstantinovic et al^14^ reported a response rate of 33% in previously untreated MBC patients. Martin et al^15^ and Palombo et al^18^ observed ORRs of 30% and 22%, respectively, in patients who had received 1 prior chemotherapy regimen. However, in a phase 2 clinical trial, a response rate of only 10% was achieved in patients who had failed 1 previous chemotherapy regimen.^16^ In another study, only 1 PR was observed in 24 patients when oral etoposide was administered as a third or fourth-line therapy in heavily pretreated MBC patients.^21^ Grade 2 or greater alopecia, nausea, and mucositis affected 80%, 53%, and 40% of the patients, respectively. The study authors concluded that oral etoposide had limited activity and may have caused severe toxicity as a third or fourth-line treatment in MBC patients. Moreover, toxic deaths were observed in two phase 2 studies using this oral etoposide regimen. In our study, oral etoposide was given at 60 mg/m^2^/d for 10 consecutive days in a 21-day cycle. We presumed that shorter exposure times and relatively extended dosing intervals would result in a quicker recovery from hematologic toxicities, especially for heavily pretreated patients. In our analysis, the current oral etoposide regimen showed a better safety profile than that of 21 days of treatment in a 28-day cycle.^22^ The most common hematologic adverse events were leukopenia (66.6%), neutropenia (60.6%), and nausea/vomiting (59.9%). Grade 3/4 nonhematological adverse events occurred in only 3 patients (vomiting, 2.6%, n = 2; alopecia, 1.3%, n = 1).

The primary endpoint of the current study was PFS. All participants received prior treatment with anthracycline and taxane-containing regimens, and approximately 50% had undergone at least 3 regimens for metastatic disease. The median PFS of the overall patient population was 4.5 months, the PR rate was 9.3%, and the CBR was 21.3%. Even in patients who had received >3 regimens for MBC, a CBR of 13.2% was observed. Survival outcomes achieved in our study were comparable to those of capecitabine monotherapy in MBC as a third-line treatment.^17,23,24^ Our results also suggested that patients who had luminal-type BC and those who had no visceral organ involvement may be the populations that benefit most from oral etoposide treatment.

This multicenter, phase 2 study demonstrated that oral etoposide was relatively well tolerated and effective in pretreated Chinese patients with MBC. Additionally, our study results provide justification for the use of oral etoposide in heavily pretreated MBC, as well as a basis for further refinement of the optimal dosing schedule.
